# Da Vinci SP radical prostatectomy: a multicentric collaboration and step-by-step techniques

**DOI:** 10.1590/S1677-5538.IBJU.2022.99.15

**Published:** 2022-04-10

**Authors:** Marcio Covas Moschovas, Isabella Brady, Abdel Rahman Jaber, Mahmoud Abou Zeinab, Aaron Kaviani, Jihad Kaouk, Simone Crivellaro, Jean Joseph, Alexandre Mottrie, Vipul Patel

**Affiliations:** 1 AdventHealth Global Robotics Institute Celebration USA AdventHealth Global Robotics Institute (GRI), Celebration, USA;; 2 University of Central Florida Orlando USA University of Central Florida (UCF), Orlando, USA;; 3 Glickman Urological & Kidney Institute Cleveland Clinic Cleveland USA Glickman Urological & Kidney Institute, Cleveland Clinic, Cleveland, USA;; 4 University of Illinois Chicago USA University of Illinois, Chicago, USA;; 5 University of Rochester New York USA University of Rochester, New York, USA;; 6 OLV Hospital ORSI Academy Aalst Belgium OLV Hospital/ ORSI Academy, Aalst, Belgium

## Abstract

**Introduction:**

Several techniques of robotic-assisted radical prostatectomy (RARP) using the da Vinci SP (SP) have been described since its clearance by the FDA (Food and Drug Administration) in 2018 ( 1 , 2 ). Even with the expanding literature about this robot, the SP technology has been restricted to a few centers in the US and Asia due to the recent release of this robot in the marked.3 In this scenario, we provided, in this video compilation, a consensus of SP referral centers describing the current approaches and techniques of da Vinci SP Radical prostatectomy (SP-RARP).

**Surgical Technique:**

We have illustrated five different techniques, including transperitoneal, extraperitoneal, Retzius-sparing, transvesical, and transperineal ( 4 - 6 ). Each surgery demonstrated crucial steps from the trocar placement until anastomosis. All approaches follow anatomic concepts and landmarks to minimize positive surgical margins, optimize oncological outcomes and promote optimal functional recovery. The trocar placement and the use of an assistant port were selected according to the operative technique of each institution. None of these surgeries had intra- or postoperative complications, and the pain management until discharge was controlled without using narcotics. All patients were discharged in less than 16 hours of surgery.

**Conclusion:**

Robotic-assisted radical prostatectomy performed with the da Vinci SP is feasible and safe with optimal perioperative outcomes. Five different approaches were described in this video compilation, and we believe that the technical details provided by this multicentric collaboration are crucial for centers willing to initiate the SP approach to radical prostatectomy.

## References

[1] Covas Moschovas M, Bhat S, Rogers T, Thiel D, Onol F, Roof S, et al. Applications of the da Vinci single port (SP) robotic platform in urology: a systematic literature review. Minerva Urol Nephrol. 2021;73:6-16. 10.23736/S2724-6051.20.03899-0 32993277

[2] Moschovas MC, Seetharam Bhat KR, Onol FF, Rogers T, Ogaya-Pinies G, Roof S, et al. Single-port technique evolution and current practice in urologic procedures. Asian J Urol. 2021;8:100-4. 10.1016/j.ajur.2020.05.003 PMC7859361 33569276

[3] Garisto J, Bertolo R, Reese SW, Bove P, Kaouk J. Minimizing minimally invasive surgery: Current status of the single-port robotic surgery in Urology. Actas Urol Esp (Engl Ed). 2021;45:345-52. English, Spanish. 10.1016/j.acuroe.2021.04.011 34088433

[4] Covas Moschovas M, Kind S, Bhat SK, Noel J, Sandri M, Rogers TP, et al. Implementing the da Vinci SP® without increasing positive surgical margins: experience and pathological outcomes of a prostate cancer referral center. J Endourol. 2021;29. Epub ahead of print. 10.1089/end.2021.0656 34963334

[5] Bassett JC, Salibian S, Crivellaro S. Single-Port Retzius-Sparing Robot-Assisted Radical Prostatectomy: Feasibility and Early Outcomes. J Endourol. 2022; 12. Epub ahead of print. 10.1089/end.2021.0542 34931527

[6] Kaouk J, Sawczyn G, Wilson C, Aminsharifi A, Fareed K, Garisto J, et al. Single-Port Percutaneous Transvesical Simple Prostatectomy Using the SP Robotic System: Initial Clinical Experience. Urology. 2020;141:173-7. 10.1016/j.urology.2020.02.024 32171697

